# Inhibitors of synaptic vesicle exocytosis reduce surface expression of postsynaptic glutamate receptors

**DOI:** 10.1080/19768354.2020.1838607

**Published:** 2020-11-03

**Authors:** Dong Ho Woo, Young-Na Hur, Minwoo Wendy Jang, C. Justin Lee, Mikyoung Park

**Affiliations:** a Drug Abuse Research Group, Research Center of Convergence Toxicology, Korea Institute of Toxicology, Daejeon, South Korea; b Brain Science Institute, Korea Institute of Science and Technology, Seoul, South Korea; c KU-KIST Graduate School of Converging Science and Technology, Korea University, Seoul, South Korea; d Center for Cognition and Sociality, Cognitive Glioscience Group, Institute for Basic Science, Daejeon, Korea; e Department of Neuroscience, Korea University of Science and Technology, Daejeon, South Korea

**Keywords:** Exocytosis blocker, AMPA receptor, NMDA receptor

## Abstract

Bafilomycin A1, a vacuolar H^+^-ATPase inhibitor, and botulinum toxin B and tetanus toxin, both vesicle fusion inhibitors, are widely known exocytosis blockers that have been used to inhibit the presynaptic release of neurotransmitters. However, protein trafficking mechanisms, such as the insertion of postsynaptic receptors and astrocytic glutamate-releasing channels into the plasma membrane, also require exocytosis. In our previous study, exocytosis inhibitors reduced the surface expression of astrocytic glutamate-releasing channels. Here, we further investigated whether exocytosis inhibitors influence the surface expression of postsynaptic receptors. Using pH-sensitive superecliptic pHluorin (SEP)-tagged postsynaptic glutamate receptors, including GluA1, GluA2, GluN1, and GluN2A, we found that bafilomycin A1, botulinum toxin B, and/or tetanus toxin reduce the SEP fluorescence of SEP-GluA1, SEP-GluA2, SEP-GluN1, and SEP-GluN2A. These findings indicate that presynaptic vesicle exocytosis inhibitors also affect the postsynaptic trafficking machinery for surface expression. Finally, this study provides profound insights assembling presynaptic, postsynaptic and astrocytic viewpoints into the interpretation of the data obtained using these synaptic vesicle exocytosis inhibitors.

## Introduction

Synaptic vesicle exocytosis is an important cellular process that releases neurotransmitters, which can initiate the delivery of synaptic information from presynaptic to postsynaptic neurons, called neurotransmission. The vacuolar H^+^-adenosine triphosphatases (ATPases) located on the synaptic vesicles acidify the synaptic vesicle interior, and the acidified synaptic vesicles are able to be loaded with neurotransmitters (Gowrisankaran and Milosevic 2020). Vacuolar H^+^-ATPase blockers, such as bafilomycin A1 and concanamycin A, inhibit neurotransmitter uptake into the synaptic vesicles and consequently the subsequent exocytosis of the synaptic vesicles as well (Floor et al. 1990; Pocock et al. 1995; Poea-Guyon et al. 2013). H^+^-ATPase is not a component of the exocytic machinery of synaptic vesicles, but it senses the pH of the synaptic vesicle interior, which modulates the exocytic vesicle fusion machinery; therefore, H^+^-ATPase contributes to synaptic vesicle exocytosis and the release of neurotransmitters.

In addition to the control by synaptic vesicle acidification through H^+^-ATPase, synaptic vesicle exocytosis can also be regulated through manipulation of the exocytic machinery molecules, which directly mediate synaptic vesicle fusion into the plasma membrane. Synaptic vesicles are fused into the plasma membrane using the soluble N-ethylmaleimide sensitive factor (NSF)-attachment protein receptor (SNARE) complex, which consists of synaptobrevin, syntaxin, and synaptosomal-associated protein 25 (SNAP-25) (Wilson et al. 1989; Sollner et al. 1993; Sudhof and Rizo 2011). Botulinum toxin B and tetanus toxin have been demonstrated to specifically cleave synaptobrevin, a component of the SNARE complex, thus blocking the exocytosis of synaptic vesicles (Link et al. 1992; Schiavo et al. 1992).

As an analogy to the neurotransmitter release mediated by presynaptic vesicle exocytosis, postsynaptic neurotransmitter receptor trafficking to the plasma membrane also requires exocytosis (Ahmad et al. 2012; Anggono and Huganir 2012; Park 2018). H^+^-ATPases are not only located on the synaptic vesicles but also on multiple acidic intracellular organelles, including endosomes, lysosomes, Golgi complexes, endocytic vesicles, exocytic vesicles, and secretory vesicles (Forgac et al. 1983; Van Dyke and Belcher 1994; Beyenbach and Wieczorek 2006; Jefferies et al. 2008; Gowrisankaran and Milosevic 2020), indicating that proper acidification of intracellular organelles and vesicles is an important process for the exocytosis of postsynaptic receptors. In addition to this, vesicle fusion into the plasma membrane, which is mediated by postsynaptic SNARE proteins, is another important step in order for postsynaptic proteins to be exocytosed (Bin et al. 2019).

Besides communication between presynapses and postsynapses for brain function, glia have been increasingly considered for their crucial contributions in brain function by constructing tripartite synapses along with presynapses and postsynapses (Araque et al. 1999; Barres 2003; Stellwagen and Malenka 2006; Lee et al., 2007; Navarrete and Araque 2008; Perea et al. 2009; Lee et al. 2010; Santello et al. 2012; Woo et al. 2012; Liddelow and Barres 2017; Farhy-Tselnicker and Allen 2018; Arizono et al. 2020). Interestingly, astrocytes release neurotransmitters, called gliotransmitters, such as glutamates and γ-aminobutyric acid (Araque et al. 2000; Perea and Araque 2007; Lee et al. 2010; Woo et al. 2012) through two glutamate-releasing channels, including a two-pore domain potassium channel, TREK-1 (Woo et al. 2012), and a Ca^2+^-activated Cl^-^ channel, Best1 (Oh et al. 2012; Park et al. 2013). These two functionally important channels in glia are also required for being trafficked to the plasma membrane by riding on the exocytic pathway.

Not only presynaptic vesicle trafficking to the plasma membrane but protein trafficking mechanisms such as the delivery of postsynaptic receptors and astrocytic glutamate-releasing channels into the plasma membrane also require exocytosis. In our previous study (Woo et al. 2012), exocytosis inhibitors reduced the surface expression of astrocytic glutamate-releasing channels. Therefore, in this study we further investigated whether conventionally used synaptic vesicle exocytosis inhibitors affect the surface expression of postsynaptic receptors. Live-cell imaging using a pH-sensitive superecliptic pHluorin (SEP) revealed that exocytosis blockers of presynaptic vesicles, such as bafilomycin A1, botulinum toxin B, and tetanus toxin, reduce the surface expression of postsynaptic glutamate receptors. To clearly rule out any interference among the presynaptic, postsynaptic, and astrocytic effects, we used a heterologous overexpression system; if neuronal cells or neuron-glia mixed cultures had been used, the data interpretation could have been complicated due to interference among presynaptic and postsynaptic neuronal and glial effects raised through treatment with the exocytosis blockers.

## Materials and methods

### Chemicals

Bafilomycin A1 was purchased from Sigma-Aldrich (MO, USA) or Tocris Bioscience (MN, USA). Tetanus toxin was purchased from Tocris Bioscience (MN, USA), and botulinum toxin B was purchased from List Biological Labs (CA, USA).

### DNA constructs

SEP-tagging glutamate receptors, including pCI-SEP-GluA1, pCI-SEP-GluA2, pCI-SEP-GluN1, and pCI-SEP-GluN2A were gifts from Robert Malinow (Addgene plasmids #24000, #24001, #23999, and #23997, respectively; note that the plasmids were deposited with the names before the new nomenclature into the Addgene: pCI-SEP-GluR1, pCI-SEP-GluR2, pCI-SEP-NR1, and pCI-SEP-NR2A, respectively) (Kopec et al. 2006).

### Cell culture and DNA transfection

Human embryonic kidney (HEK) 293 T cells were grown in Dulbecco’s modified Eagle’s medium (DMEM, Invitrogen) supplemented with 10% fetal bovine serum (Invitrogen).

For live-cell imaging, pCI-SEP-GluA1, pCI-SEP-GluA2, pCI-SEP-GluN1 or pCI-SEP-GluN2A plasmids were transfected into HEK 293 T cells using Lipofectamine 2000 (Invitrogen) and were expressed for 24–36 h. For the cell-surface biotinylation assay, SEP-GluA1 plasmids were transfected into HEK 293 T cells using Effectene (Qiagen) and expressed for 24–36 h.

### Live-cell imaging

HEK 293 T cells overexpressing SEP-GluA1, SEP-GluA2, SEP-GluN1, or SEP-GluN2A, which had been grown on the coverslip, were transferred to an imaging chamber (Live Cell Instrument, Seoul, Korea) filled with imaging solution (mM: 120 NaCl, 3 KCl, 2 CaCl_2_, 2 MgCl_2_, 15 glucose, 15 HEPES, pH 7.35). The cells were live-imaged confocally every 15 s for 7–8 min before the appropriate drug applications, and then live-cell imaging was continued every 15 s for the next 35 min in the presence of the drugs. Confocal imaging was performed using the Revolution XD System (Andor Technology) equipped with a Yokogawa CSU-X1 spinning disk confocal unit, a 488 nm solid-state laser, a 60× Plan Apochromat objective (NA 1.4), and a 14-bit iXON3 DU-885 EMCCD camera (Andor Technology). Using the Metamorph imaging and analysis software program (Molecular Device Inc.), the complete confocal z-sectioned images were acquired, and maximum intensity projected images were produced.

### Image analysis and quantification

To analyze SEP fluorescence, the integrated intensity of SEP-GluA1, SEP-GluA2, SEP-GluN1, or SEP-GluN2A from a single HEK 293 T cell was measured using the Metamorph software. To calculate the changes in SEP fluorescence intensity (ΔF/F_0_), the change in fluorescence intensity (ΔF) was normalized to F_0_, where ΔF was calculated by F_t_−F_0_, in which F_t_ and F_0_ indicate the intensity at each time point and the average intensity of all time points prior to drug treatment, respectively.

### Cell-surface biotinylation assay, including immunoblot analysis

HEK 293 T cells overexpressing SEP-GluA1 were washed three times with PBS, and cell surface-expressed proteins were biotinylated in PBS containing 1 mg/ml Ez-Link Sulfo-NHS-LC-biotin (Thermo Scientific) for 30 min. Cells were washed with quenching buffer (100 mM glycine in PBS) to remove excess biotin and subsequently washed with PBS three times. Cells were then harvested in RIPA lysis buffer (Rockland) containing protease inhibitor cocktail and incubated with high capacity NeutrAvidin-Agarose Resin (Thermo Scientific) for 2 h at 4°C. After three washes with the lysis buffer, bound proteins were eluted by the SDS sample buffer and subjected to immunoblot analysis using anti-GFP (1:500, ab13970, Abcam). Protein bands on immunoblots were visualized by a chemiluminescence method (BioRad) and an imaging documentation system (ImageQuant LAS 500, GE Healthcare) and quantified using Image*J* software (National Institutes of Health, http://rsbweb.nih.gov/ij/).

## Results

### Inhibitors of synaptic vesicular exocytosis reduced the surface expression of neuronal AMPA receptors

**Figure 1.**
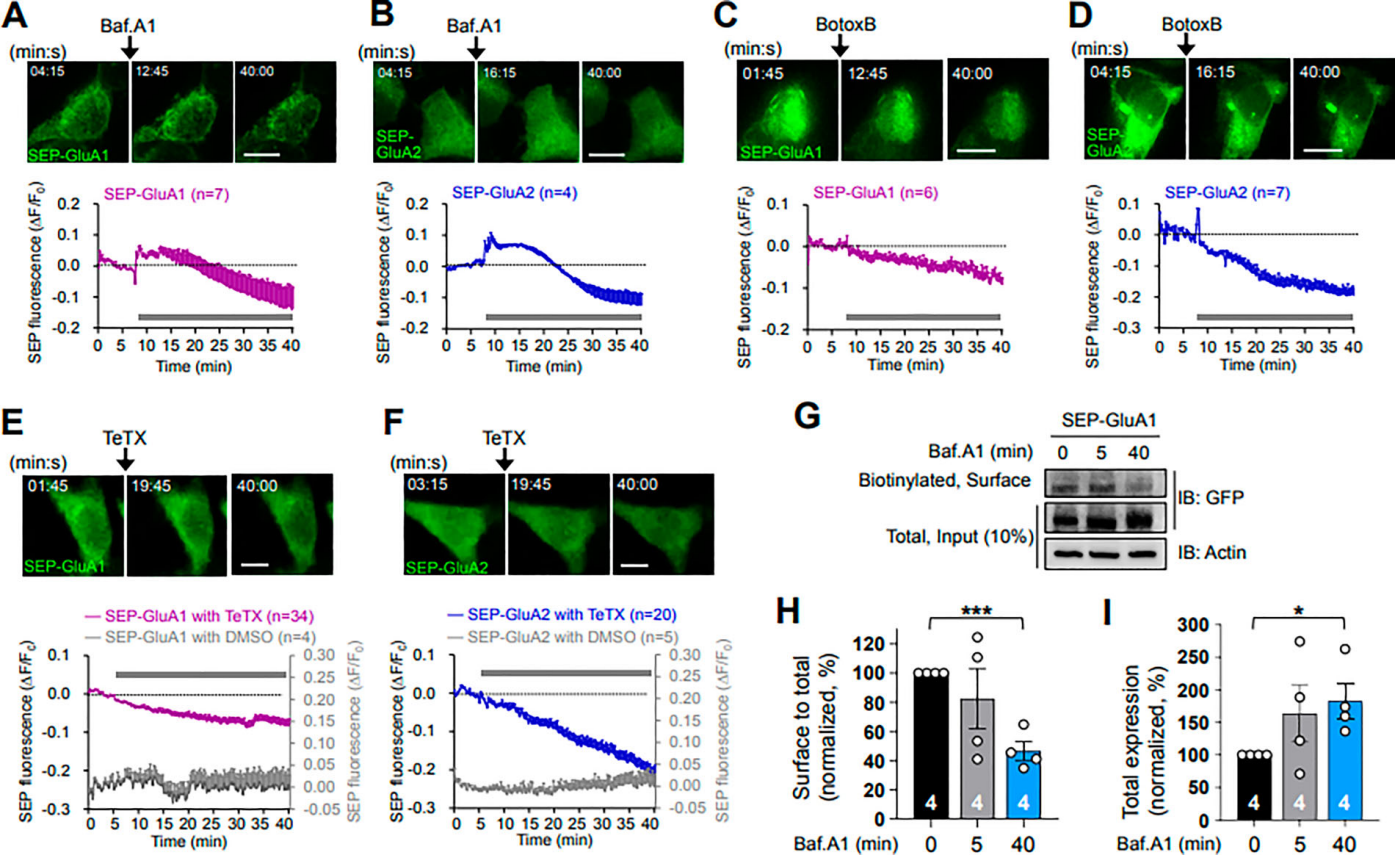
Bafilomycin A1, botulinum toxin B, and tetanus toxin reduce the surface expression level of α-amino-3-hydroxy-5-methyl-4-isoxazolepropionate (AMPA) receptors. (A and B) (Upper) Representative confocal images obtained from the live-cell imaging of HEK 293 T cells overexpressing SEP-GluA1 (A) or SEP-GluA2 (B) before and after bafilomycin A1 (Baf.A1, 2 μM) treatment. Scale bars, 10 μm. (Lower) Data represent the means ± SEM of the changes in SEP fluorescence intensity (ΔF/F_0_). F_0_, the average intensity of all time points prior to drug treatment; ΔF = F_t_−F_0_; F_t_, the intensity at each time point. Gray bars, the period of Baf.A1 applied. Note the increases in SEP fluorescence during the early period following Baf.A1 treatment and the subsequent gradual reductions in fluorescence. (C and D) (Upper) Representative confocal images obtained from the live-cell imaging of HEK 293 T cells overexpressing SEP-GluA1 (C) or SEP-GluA2 (D) before and after botulinum toxin B (BotoxB, 8 μg/ml) treatment. Scale bars, 10 μm. (Lower) Data represent the means ± SEM of the changes in SEP fluorescence intensity (ΔF/F_0_). Gray bars, the period of BotoxB applied. Note the gradual reductions in SEP fluorescence immediately after BotoxB treatment. (E and F) (Upper) Representative confocal images obtained from the live-cell imaging of HEK 293 T cells overexpressing SEP-GluA1 (E) or SEP-GluA2 (F) before and after tetanus toxin (TeTX, 4 μg/ml) treatment. Scale bars, 10 μm. (Lower) Data represent the means ± SEM of the changes in SEP fluorescence intensity (ΔF/F_0_). Gray bars, the period of TeTX applied. Note the gradual reductions in SEP fluorescence immediately after TeTX treatment. (G − I) Surface expression levels of SEP-GluA1 by the Baf.A1 (2 μM) treatment were evaluated using a cell-surface biotinylation assay from the HEK 293 T cells overexpressing SEP-GluA1. Representative immunoblots are shown (G). Data represent the means ± SEM of the surface-to-total expression levels (H) and the total expression levels (I) of SEP-GluA1. *p < 0.05, ***p < 0.0005 as indicated, Student’s *t* test.

**Figure 2.**
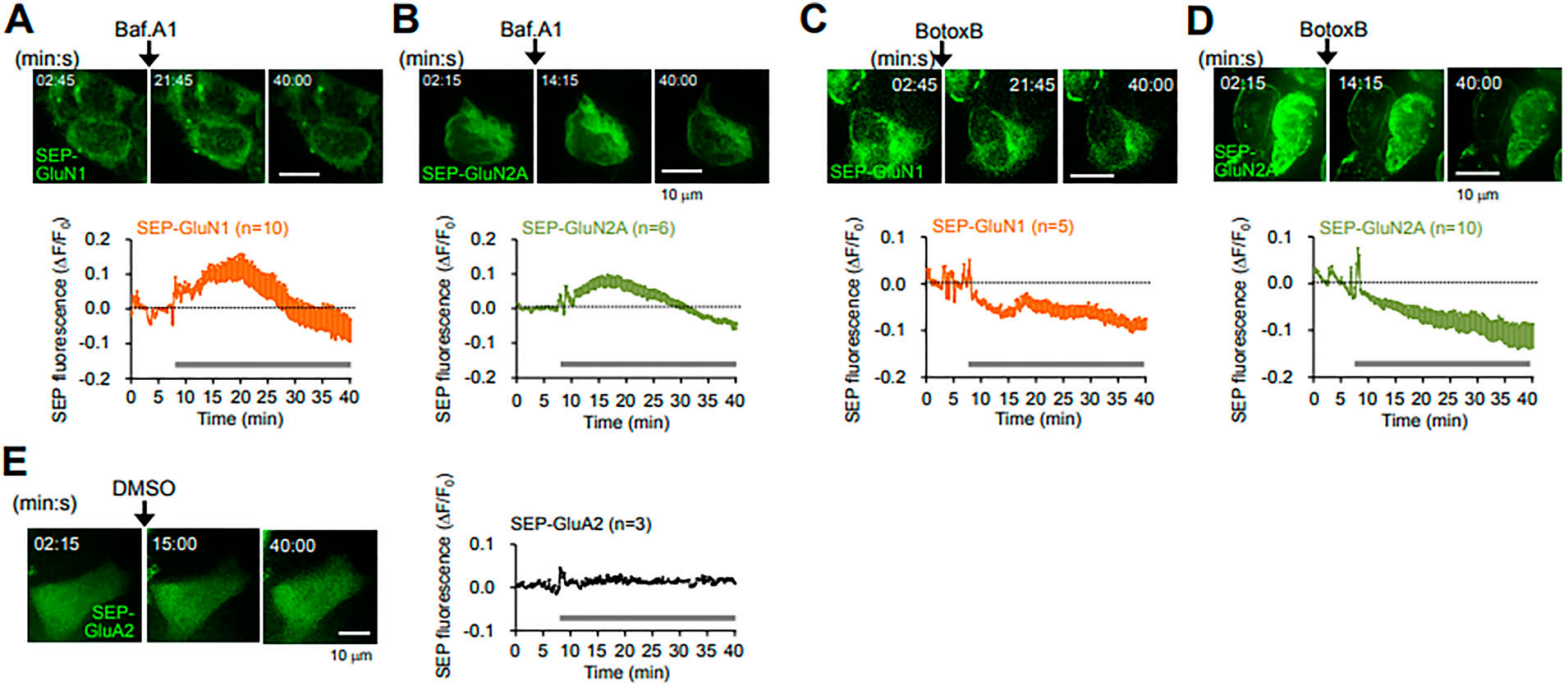
Bafilomycin A1 and botulinum toxin B reduce the surface expression level of N-methyl-D-aspartate (NMDA) receptors. (A and B) (Upper) Representative confocal images obtained from the live-cell imaging of HEK 293 T cells overexpressing SEP-GluN1 (A) or SEP-GluN2A (B) before and after bafilomycin A1 (Baf.A1, 2 μM) treatment. Scale bars, 10 μm. (Lower) Data represent the means ± SEM of the changes in SEP fluorescence intensity (ΔF/F_0_). F_0_, the average intensity of all time points prior to drug treatment; ΔF = F_t_−F_0_; F_t_, the intensity at each time point. Gray bars, the period of Baf.A1 applied. Note the increases in SEP fluorescence during the early period following Baf.A1 treatment and the subsequent gradual reductions in fluorescence. (C and D) (Upper) Representative confocal images obtained from the live-cell imaging of HEK 293 T cells overexpressing SEP-GluN1 (C) or SEP-GluN2A (D) before and after botulinum toxin B (BotoxB, 8 μg/ml) treatment. Scale bars, 10 μm. (Lower) Data represent the means ± SEM of the changes in SEP fluorescence intensity (ΔF/F_0_). Gray bars, the period of BotoxB applied. Note the gradual reductions in SEP fluorescence immediately after BotoxB treatment. (E) (Left) Representative confocal images obtained from the live-cell imaging of HEK 293 T cells overexpressing SEP-GluA2 before and after DMSO treatment. Scale bar, 10 μm. (Right) Data represent the means ± SEM of the changes in SEP fluorescence intensity (ΔF/F_0_). Gray bar, the period of DMSO applied.

**Figure 3.**
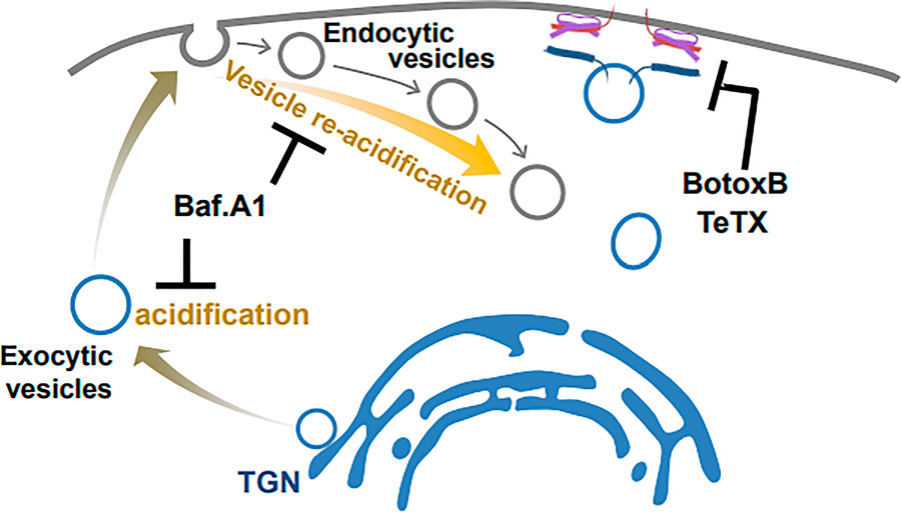
A model showing that the conventional inhibitors of synaptic vesicle exocytosis affect the exocytosis of postsynaptic AMPA and NMDA receptors. A vacuolar H^+^-ATPase inhibitor, Baf.A1, blocks the functional surface expression of neuronal postsynaptic AMPA and NMDA receptors. In addition, vesicle fusion blockers, such as BotoxB and TeTX, inhibit the functional surface expression of neuronal postsynaptic AMPA and NMDA receptors. For details, see the main text.

We first investigated whether the conventional tools such as bafilomycin A1, botulinum toxin B, or tetanus toxin, which are known to inhibit synaptic vesicle exocytosis, affect the surface expression of postsynaptic AMPA receptors, which are normally mediated by vesicle exocytosis. To selectively detect the surface receptors, we utilized a pH-sensitive variant of GFP, SEP-tagging AMPA receptors (SEP-GluA1 and SEP-GluA2 subunits), and N-methyl-D-aspartate (NMDA) receptors (SEP-GluN1 and SEP-GluN2A subunit), since their fluorescent signals are quenched when exposed to an acidic environment, such as the lumen of an intracellular vesicle or organelle, and they are visible when exposed to an extracellular neutral pH environment (Kopec et al. 2007; Wang et al. 2008; Kennedy et al. 2010; Cho et al. 2015). SEP signals from SEP-GluA1 and SEP-GluA2 expressed in HEK 293 T cells were initially enhanced for 10 and 15 min, respectively, immediately following bafilomycin A1 treatment (2 μM), compared with the SEP signals obtained prior to bafilomycin A1 treatment at the basal levels, and then they gradually decreased until the imaging ended (Figure 1A and B). Cell-surface biotinylation assay to detect the surface-expressed receptors (Suh et al. 2010) revealed that the surface-to-total expression level of SEP-GluA1 was not altered by a 5-min treatment with bafilomycin A1 (2 μM), where the SEP fluorescence was increased in the live-cell imaging (Figure 1A), compared to untreated control (0-min) (Figure 1G and H). These data together support that the initial enhancement of SEP signals induced by bafilomycin A1 treatment during the live-cell imaging was not due to an actual physical increase in the receptors on the surface. The initial enhancement in the SEP signals of SEP-GluA1 and SEP-GluA2 could be due to the inhibition of the re-acidification of endocytosed vesicles containing SEP-GluA1 and SEP-GluA2 during the bafilomycin A1 treatment and to the SEP-GluA1- and SEP-GluA2-containing recycling vesicles that were initially available in the low pH environment of the lumen before the bafilomycin A1 treatment. SEP-GluA1 and SEP-GluA2 on these initially available recycling vesicles are likely exocytosed to the surface and subsequently endocytosed during the bafilomycin A1 treatment (Figure 3). The initial enhancements of SEP-GluA1 for 10 min and SEP-GluA2 for 15 min suggest that the trafficking rate between surface and intracellular compartments might be faster in the GluA2 than in the GluA1 subunit. Further reductions of SEP signals in SEP-GluA1 (Figure 1A and B) and SEP-GluA2 (Figure 2A and B) far below the basal levels and the significant reduction in the surface-to-total expression level of SEP-GluA1 by the 40-min treatment of bafilomycin A1 compared to untreated control (0-min) (Figure 1G and H) suggest that bafilomycin A1 treatment reduces the surface expression of GluA1 and GluA2, most likely by reducing exocytosis of secretory vesicles containing GluA1 and GluA2 and the fusion of just-endocytosed vesicles containing GluA1 and GluA2 into the intracellular organelles that existed under the low pH environment in the lumen before bafilomycin A1 treatment. No change and an increase in the total expression of SEP-GluA1 under the 5- and 40-min bafilomycin A1 treatment, respectively (Figure 1I) further support that the decrease of SEP signals by bafilomycin A1 treatment is due to the reduction of surface-existing receptors and not due to the degradation of SEP-conjugated receptors.

Treatment with inhibitors that block the exocytic vesicle fusion step, such as botulinum toxin B (8 μg/ml; Figure 1C and D) or tetanus toxin (4 μg/ml; Figure 1E and F), gradually reduced the SEP signals of SEP-GluA1 and SEP-GluA2 immediately after the drug treatment. Considering the larger reduction of the SEP signal in SEP-GluA2 compared with that in SEP-GluA1, the trafficking rate between surface and intracellular compartments seems far faster in the GluA2 than in the GluA1 subunit. To rule out the possibility that the gradual reduction of SEP signals might be caused by photo-bleaching during live-cell imaging, the SEP signals from SEP-GluA1 and SEP-GluA2 remained consistent without drug treatment during the whole period of live-cell imaging (gray graphs, Figure 1E and F).

### Inhibitors of synaptic vesicular exocytosis reduced the surface expression of neuronal NMDA receptors

Next, we tested whether the surface expression of NMDA receptors, another major postsynaptic glutamate receptor, is also affected by treatment with drugs known to inhibit synaptic vesicle exocytosis. Using SEP-tagging NMDA receptor subunits such as SEP-GluN1 and SEP-GluN2A, we found that the SEP signals of SEP-GluN1 (Figure 2A) and SEP-GluN2A (Figure 2B) are, similar to GluA1 and GluA2 (Figure 1), also initially increased. This increase is likely due to the inhibition of the re-acidification of endocytosed vesicles containing SEP-GluN1 and SEP-GluN2A during the bafilomycin A1 treatment and to the initially available recycling pool of SEP-GluN1 and SEP-GluN2A under the low luminal pH before bafilomycin A1 treatment, which are readily exocytosed to the surface and subsequently endocytosed during the bafilomycin A1 treatment (Figure 3). In addition, the SEP signals of SEP-GluN1 and SEP-GluN2A were reduced below the basal levels from around 20 and 22 min after bafilomycin A1 treatment, respectively, indicating that bafilomycin A1 reduces the surface expression of GluN1 and GluN2A, probably by reducing the exocytosis of secretory vesicles containing GluN1 and GluN2A and by the fusion of just-endocytosed vesicles with the intracellular organelles that existed under the low pH environment in the lumen before bafilomycin A1 treatment. Treatment with botulinum toxin B (8 μg/ml) gradually reduced the SEP signals of SEP-GluN1 and SEP-GluN2A immediately after the drug treatment (Figure 2C and D), indicating that botulinum toxin B treatment reduces the surface expression of GluN1 and GluN2A. As a control, the SEP signals were kept consistent without drug treatment (Figure 2E), indicating that the reduction of SEP signals was not due to photo-bleaching during the live-cell imaging period.

Considered together, bafilomycin A1 transiently increased and subsequently decreased the surface expression of AMPA (Figure 1A and B) and NMDA receptors (Figure 2A and B), suggesting that bafilomycin A1 not only inhibits further trafficking of secretory exocytic vesicles through inhibiting acidification, but also seems to inhibit the re-acidification of AMPA and NMDA receptor-containing endocytosed vesicles (Figure 3). Botulinum toxin B also decreased the surface expression of AMPA (Figure 1C and 2D) and NMDA receptors (Figure 2C and D) by inhibiting the fusion of exocytic vesicles containing AMPA and NMDA receptors into the plasma membrane (Figure 3).

## Discussion

In the present study, we demonstrated that conventional synaptic vesicle exocytosis inhibitors, such as bafilomycin A1, botulinum toxin B, and tetanus toxin, block the surface expression of postsynaptic AMPA and NMDA receptors. Specifically, a vacuolar H^+^-ATPase inhibitor, bafilomycin A1 blocks the functional surface expression of neuronal postsynaptic AMPA and NMDA receptors, probably through the retarded acidification of the lumens of intracellular secretory exocytic vesicles and organelles (Figure 3). Vesicle fusion blockers, such as botulinum toxin B and tetanus toxin, also inhibit the functional surface expression of neuronal postsynaptic AMPA and NMDA receptors probably through the cleavage of a component of the vesicle fusion machinery (Figure 3). These findings directly evidence that synaptic vesicle exocytosis shares a mechanism with the exocytic trafficking of postsynaptic glutamate receptors.

In our previous study, intracellular injections of two different drugs, botulinum toxin B and tetanus toxin, which inhibit exocytic vesicle fusion by acting on the same target sites, showed different results in glutamate-induced GluA1-mediated currents (Woo et al. 2012); botulinum toxin B (8 μg/ml, 20 min) did not significantly reduce the GluA1-mediated currents whereas tetanus toxin (4 μg/ml, 20 min) significantly reduced those currents (Woo et al. 2012). The possibilities could not be ruled out that tetanus toxin may act more efficiently than botulinum toxin B under the experimental condition of a 20-min injection time, and/or that an 8 μg/ml concentration of botulinum toxin B (Woo et al. 2012) may not be appropriate to block the surface delivery of GluA1s. Therefore, it would be interesting to investigate whether the efficacies of such drugs differ based on the experimental parameters, such as the duration of application, concentration, types of stimuli, brain cell types, and so on. In addition, it would be interesting to further study whether the molecular identities of exocytic vesicles display delicate acting mechanisms mediated by these drugs.

We utilized SEP to visualize the surface-located AMPA and NMDA receptors (Figure 1 and 2). However, using SEP and bafilomycin A1 at the same time to detect the surface receptors provides rather complicated interpretations (Sankaranarayanan and Ryan 2000). SEP is a pH-sensitive fluorophore whose fluorescence is quenched in acidic environments, and bafilomycin A1 is an acidification inhibitor, which acts on V-ATPase in vesicles. In the presence of bafilomycin A1, which inhibits the luminal re-acidification of just-endocytosed vesicles (Contreres et al. 1998; Samms et al. 1999) as well as that of vesicles on the exocytic secretory pathway (Palokangas et al. 1994; Zoccarato et al. 1999), SEP fluorescence includes surface receptors along with parts of intracellular vesicles. Thus, the initial increases in the SEP signals of SEP-GluA1, SEP-GluA2, SEP-GluN1, and SEP-GluN2A with bafilomycin A1 treatment (Figure 1A, 1B, 2A, and 2B) were mainly attributed to SEP-receptors on the surface as well as those on the just-endocytosed vesicles. Further gradual reductions in SEP fluorescence after the initial enhancement (Figure 1A, 1B, 2A, and 2B) could have resulted from the depletion of vesicles to be exocytosed in the secretory pathway due to the bafilomycin A1-mediated inhibition of vesicle luminal acidification and the fusion of just-endocytosed vesicles with intracellular organelles that existed under the low pH environment in the lumen before bafilomycin A1 treatment. Unlike bafilomycin A1, botulinum toxin B and tetanus toxin, which are vesicle fusion blockers, can act on all vesicles, including the endocytic recycling pool of vesicles that were exocytosed to the plasma membrane (Figure 3).

Interestingly, our data showing a slower initial increase in SEP signals of NMDA receptors (Figure 2A and B) compared to the signals of AMPA receptors (Figure 1A and B) following bafilomycin A1 treatmen, imply the involvement of differential molecular machinery in AMPA and NMDA receptor trafficking, including endocytosis, endocytic recycling, and exocytosis, in HEK 293 T cells, similar to that in neuronal cells. We cannot also rule out that the differential regulation between AMPA and NMDA receptor trafficking in HEK 293 T cells might be due to the different intrinsic channel properties of AMPA and NMDA receptors, which could endow their own trafficking mechanisms.

In conclusion, the present study reassessed the effects of inhibitors conventionally and widely used to block synaptic vesicle exocytosis, and it demonstrated that the synaptic vesicle exocytosis inhibitors block the exocytosis of postsynaptic glutamate receptors. Therefore, along with the previous study (Woo et al. 2012) showing that exocytosis inhibitors reduced the surface expression of astrocytic glutamate-releasing channels, this study potentially provides more expansive and profound insights assembling presynaptic, postsynaptic, and astrocytic points of view, into the interpretation of the data obtained using these inhibitors in the field.
